# A high-quality genome assembly of the waterlily aphid *Rhopalosiphum nymphaeae*

**DOI:** 10.1038/s41597-024-03043-3

**Published:** 2024-02-13

**Authors:** Yangzi Wang, Shuqing Xu

**Affiliations:** 1 Institute of Organismic and Molecular Evolution (iomE), Johannes Gutenberg University Mainz, 55128 Mainz, Germany; 2 Institute for Evolution and Biodiversity, University of Münster, 48161 Münster, Germany

**Keywords:** Genome, Entomology

## Abstract

Waterlily aphid, *Rhopalosiphum nymphaeae* (Linnaeus), is a host-alternating aphid known to feed on both terrestrial and aquatic hosts. It causes damage through direct herbivory and acting as a vector for plant viruses, impacting worldwide *Prunus* spp. fruits and aquatic plants. Interestingly, *R. nymphaeae*’s ability to thrive in both aquatic and terrestrial conditions sets it apart from other aphids, offering a unique perspective on adaptation. We present the first high-quality *R. nymphaeae* genome assembly with a size of 324.4 Mb using PacBio long-read sequencing. The resulting assembly is highly contiguous with a contig N50 reached 12.7 Mb. The BUSCO evaluation suggested a 97.5% completeness. The *R. nymphaeae* genome consists of 16.9% repetitive elements and 16,834 predicted protein-coding genes. Phylogenetic analysis positioned *R. nymphaeae* within the Aphidini tribe, showing close relations to *R. maidis* and *R. padi*. The high-quality reference genome *R. nymphaeae* provides a unique resource for understanding genome evolution in aphids and paves the foundation for understanding host plant adaptation mechanisms and developing pest control strategies.

## Background & Summary

**Fig. 1:**
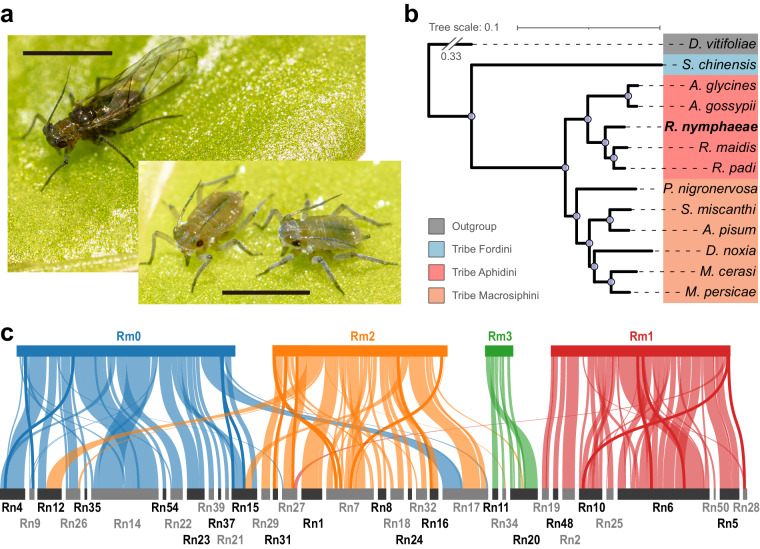
Evolution of *R. nymphaeae*. (**a**) Pictures show *R. nymphaeae* alatae (up left) and apterae (down right) feeding on the great duckweed [*Spirodela polyrhiza* (L.) Schleid., Araceae]. Black scale bars indicate 1 mm. (**b**) The plot shows the Maximum-likelihood phylogenomic tree reconstructed based on the one-to-one orthologs of 12 aphids (*Schlechtendalia chinensis*, *Aphis gossypii*, *Aphis glycines*, *Rhopalosiphum nymphaeae*, *Rhopalosiphum maidis*, *Rhopalosiphum padi*, *Pentalonia nigronervosa*, *Sitobion miscanthi*, *Acyrthosiphon pisum*, *Diuraphis noxia*, *Myzus cerasi*, and *Myzus perisicae*) and the grape phylloxera (*Daktulosphaira vitifoliae*) as the outgroup. The blue dots on the internal nodes indicate 100% bootstrapping support. (**c**) Genome synteny analysis between *R. nymphaeae* and *R. maidis* genomes. The up panel bars show four assembled chromosomes of *R. maidis* with their names above, while the down panel shows 36 long contigs (with lengths greater than 1 Mb) of *R. nymphaeae* with names below.

*Rhopalosiphum nymphaeae* (Linnaeus), also known as the waterlily aphid, is a polyphagous host-alternating aphid that has been reported to feed on both terrestrial hosts plants like *Prunus* spp.^1^ and various aquatic hosts belonging to Nympahaeaceae, Araceae etc.^2^ (Fig. 1a). As *R. nymphaeae* is a devastating pest through direct herbivory and as plant virus vectors to some domesticated fruits and crops^2^, the comprehensive understanding of this insect is of great agricultural value. On the other hand, *R. nymphaeae* has a contrasting host range compared to its closely related species—likely the only aphid to live in both aquatic and terrestrial conditions^2^—making it a distinctive study model for revealing how insects adapt to diverse hosts.

Here, we report the first high-quality draft genome assembly of *R. nymphaeae*, generated using PacBio long-read sequencing (~31.7 Gb HiFi reads, with N50 = 19.3 kb). After assembling long reads into contigs, we removed bacterial comtaminations (298 contigs comprising 19.5 Mb; see Supplementary Fig. 1 and Supplementary Data 1). Among them, 145 contigs matched the well-studied aphids’ endosymbiotic bacterium, *Buchnera aphidicola*^3,4^ (Supplementary Data 1). The final monoploid genome assembly of *R. nymphaeae* consists of 91 contigs with a total size of 324.4 Mb (Table 1). The contig N50 reaches 12.7 Mb, and the longest contig is 47.9 Mb (Table 1). These data suggest the contiguity of the *R. nymphaeae* genome assembly is one of the highest compared to 13 previously published aphid genomes^5–12^ (Supplementary Data 2). We identified 54.8 Mb repetitive elements, which account for 16.9% of the *R. nymphaeae* assembly (Table 2). After soft-masking the *R. nymphaeae* genome, we predicted 16,834 protein-coding genes with an average length of 6,760 bp (Table 3) using the BRAKER pipeline^13–18^ that incorporated empirical evidence of transcripts assembled from short-reads sequencing (RNA-seq) data and full-length transcripts from long-read PacBio sequencing (Iso-seq) data, and extrinsic evidence based on the homology from other aphids (see methods).

**Table 1:** *R. nymphaeae* genome assembly statistics.

Parameters	Value
Contigs_count	91
N50	12.67 Mb
N90	3.0 Mb
L50	7
L90	25
Longest_contig	47.89 Mb
Shortest_contig	0.023 Mb
GC_content	27.25%
Total_size	324.40 Mb

**Table 2:** Summary of the repetitive elements identified from the *R. nymphaeae* genome assembly.

Repeat group	Count	Length (Kb)	Coverage of genome (%)
Long interspersed nuclear elements	1,251	513.1	0.16
Long terminal repeat	180	27.0	0.01
DNA transposons	2,091	428.3	0.13
Helitron	431	184.2	0.06
Low complexity	41,749	2,097.5	0.65
Simple repeat	295,202	13,737.5	4.23
rRNA	74	589.2	0.18
Unclassified	64,425	37,215.6	11.47
Total	405,403	54,792.5	16.89

**Table 3:** Brief summary of protein-coding gene prediction in the *R. nymphaeae* genome assembly.

Sequence type	Count	Mean size (bp)
Protein-coding genes	16,834	6,760
Exons	90,923	238
Introns	74,092	1,243
3′-utr	2,530	304
5′-utr	3,490	258

We constructed a maximum likelihood phylogenetic tree based on the low-copy (often referred as single-copy) orthologs to determine the relationship of *R. nymphaeae* with the other 11 members from Aphidoidea (Fig. 1b). In accordance with the previously constructed phylogeny based on the mitochondrial sequences^19^, *R. nymphaeae* is positioned within the Aphidini tribe. It is closely related to *R. maidis* and *R. padi* (Fig. 1b).

We conducted a genome synteny analysis between *R. nymphaeae* and *R. maidis*^20^ (Fig. 1c). Despite observing several genomic rearrangements, there is a notable conservation between the two genomes. Among the 38 longest contigs (lengths greater than 1 Mb) from the *R. nymphaeae* genome, 36 exhibited synteny with four chromosomes of *R. maidis*. Most chromosomal regions from the *R. maidis* genome aligned with the *R. nymphaeae* genome assembly.

This study presents the first genome assembly for *R. nymphaeae*, providing a valuable dataset for understanding genome evolution in aphids. This genome assembly not only serves as a crucial resource for exploring potential pest control strategies, but also paves the way for subsequent comparative genomics and experimental evolution studies, aiming to decipher the adaptive mechanisms of this organism to a changing environment.

## Methods

### Sample preparation and sequencing

The aphid was collected in the summer of 2020 on a duckweed population growing near the University of Münster, Germany. A population derived from a single aphid individual was maintained in the lab on *Spirodela polyrhiza* plants. We extracted DNA from the aphids using the Monarch HMW DNA Extraction Kits. The DNA was sequenced on a Pacbio sequel II at Novogene, Beijing, China. To assist the protein-coding gene prediction, we generated both PacBio Iso-seq (27.2 Gb, N50 = 2,191 bp) and Illumina short-reads RNA-seq libraries (150 bp paired-end, 41.9 million reads) using total RNAs from the whole body of *R. nymphaeae*.

### Genome assembly and contamination screening

We assembled the genome using Hifiasm (v.0.19.3-r572)^21,22^ with high-quality HiFi reads. We trimmed both ends of reads by 20 bp (with -z20 option). Next, the assembled genome was screened using two strategies to eliminate contamination from potential sequencing adaptors and foreign DNA: the NCBI Foreign Contamination Screen (FCS) tool suite^23^ and BlobTools (v 1.1.1)^24^. For the FCS-adaptor (v 0.5.0)^23^ screening, default settings were used, and no adapter sequence was found in the assembly. Both FCS-GX (v 0.5.0)^23^ and BlobTools (v 1.1.1)^24^ identified foreign DNA, which mostly originated from bacteria. Contaminated contigs identified by FCS-GX (v 0.5.0)^23^ or BlobTools (v 1.1.1)^24^ were removed from the assembly. In the case of screening using Blobtools^24^, assembly contigs longer than 1 Mb were split into smaller fragments of 1 Mb each (with a 1 Kb overlap between two consecutive fragments) to reduce the computational burden during alignment against the UniProt Reference Proteomes^25^ using Diamond (v2.1.8.16)^26^.

### Repetitive element annotation

RepeatModeler (v2.0.2)^27^ was used to generate a *de novo* repeat library from the *R. nymphaeae* genome. The “-LTRStruct” flag was added in this step to also identify long terminal repeat structure. Next, based on the classified repeat library generated from the RepeatModeler, RepeatMasker (v4.1.5) was used to predict and soft mask the repeats in the *R. nymphaeae* genome.

### Protein-coding gene annotation

We used the BRAKER^13–18^ pipeline for protein-coding gene prediction, which combines gene models predicted based on short-read RNA-seq transcriptome (BRAKER1 method^15,28–32^) and protein homologs from other aphids (BRAKER2 method^17,28,29,33–37^). We then used TSEBRA^18^ to compare these predictions with full-length transcripts derived from Iso-seq data, ultimately selecting the most optimal gene models.

For the BRAKER1 run, paired-end RNA-seq reads from *R. nymphaeae* were processed using Trimmomatic (v0.39)^38^ with parameters of “ILLUMINACLIP:TruSeq 3-PE-2.fa:2:30:10 SLIDINGWINDOW:4:15 MINLEN:36 HEADCROP:10”, which trimmed the first 10 bp and to filter the possible Illumina sequencing adaptor sequences. FastQC (v0.11.9, https://www.bioinformatics.babraham.ac.uk/projects/fastqc/) was used to perform the quality control before and after the filtration. Next, the cleaned reads were aligned to the *R. nymphaea* genome using HISAT2 (v2.2.1)^39^ with default settings. After that, BRAKER1 was fed with the repeat soft-masked *R. nymphaea* genome and RNA-seq aligned BAM file. It automatically performed GeneMark-ET training using spliced alignment information from the RNA-seq and, based on which, predicted gene models using AUGUSTUS^40^. For the BRAKER2 run, similar automatic training of GeneMark-EP+^37^ was done to guide the AUGUSTUS’s gene prediction, but this time, BRAKER2 utilised protein-coding exon boundaries information, which was from the alignment of the protein sequences from Aphidoidea that were downloaded from UniProt^41^. For the Iso-seq data processing, the command-line tools from SMRT Link software from PacBio (https://www.pacb.com/) were used. In brief, consensus sequences generated from raw subread were filtered to remove primers, concatemers and poly(A) tails to get Full-Length Non-concatemer (FLNC) reads. These FLNC reads were then clustered, aligned to the *R. nymphaea* reference genome using minimap2 (v2.24)^42^ and collapsed using Cupcake (v0.1.4, https://github.com/Magdoll/cDNA_Cupcake) to get the full-length transcripts. GeneMarkS-T^43^ was then used to predict the protein-coding region for each full-length transcript. The gene models predicted independently from BRAKER1 and BRAKER2 were then merged and compared with full-length transcripts from Iso-seq data using TSEBRA with default options. Only the longest isoform was kept for each gene model.

After the best gene models were selected by TSEBRA, we adopted AGAT (v0.8.0, https://github.com/NBISweden/AGAT) for three rounds of filtration, including the removal of 1) genes with length less than 100 bp, 2) genes with coding sequences harbour repetitive elements higher than 20%, 3) genes have only one exon and don’t have a complete start or/and stop codon predicted.

For the functional annotation, proteins sequences translated from the gene annotation were aligned to the UniProtKB^41^ database using Blastp (BLAST + v2.12.0)^29^ with “-evalue 1e-6 -max_hsps 1 -max_target_seqs. 1 -outfmt 6” parameters and processed using InterProScan (v5.63–95.0)^44^ with “-goterms -iprlookup” options, respectively.

### Phylogenetic tree reconstruction and genome synteny analyses

We identified 3,550 low-copy (often referred as single-copy) ortholog groups based on protein sequences (translated from the longest isoform of each gene) from 12 aphid genomes, including *Schlechtendalia chinensis*^12^, *Aphis gossypii*^8^, *Aphis glycines*^10^, *R. nymphaeae*, *Rhopalosiphum maidis*^20^, *Rhopalosiphum padi*^45^, *Pentalonia nigronervosa*^9^, *Sitobion miscanthi*^7^, *Acyrthosiphon pisum*^11^, *Diuraphis noxia*^5^, *Myzus cerasi*^9^, and *Myzus perisicae*^11^, and the grape phylloxera (*Daktulosphaira vitifoliae*)^46^ genome using OrthoFinder (v2.5.4)^47,48^. Those low-copy ortholog groups were concatenated and aligned automatically by OrthoFinder and generated a multiple sequence alignment file, which was used for phylogenetic analysis. For the phylogenetic tree reconstruction, ModelTest-NG (v0.2.0)^49^ was used first and found “JTT + I + G4” to be the best model, which was later used in the maximum likelihood phylogenetic tree reconstruction using RAxML-NG (v1.2.0)^50^. We used iTOL (v6)^51^ for tree visualization.

For the genome synteny analysis, the one-to-one orthologs between *R. nymphaeae* and *R. maidis* genomes were extracted from OrthoFinder’s result and fed to MCScanX_h^52^, which was used with “-b 2” option to get the inter-species collinearity between *R. nymphaeae* and *R. maidis*. SynVisio^53^ was used to visualize the genome synteny.

### Evaluations of *R. nymphaeae* genome assembly and protein-coding gene annotation

Merqury (v1.3)^54^, an assembly evaluation software that compares the distribution of k-mers in sequencing reads and the final assembly, was used to estimate the base-level accuracy and completeness of the *R. nymphaeae* assembly, with an estimated optimal k-mer size of 19. Benchmarking Universal Single-Copy Orthologs (BUSCO, v5.4.3)^55^ was used to evaluate the genome assembly and protein-coding gene annotation of *R. nymphaeae* with “-m genome” and “-m proteins”, respectively. The “hemiptera_odb10” reference lineage database (2,510 BUSCOs) was chosen for both runs. In addition, DOGMA (v3.7)^56,57^ with “insects” reference core set was also used to assess the completeness of gene annotation in *R. nymphaeae* genome based on conserved protein domains. For the gene model structure visual checking, JBrowse 2^58^ was used.

## Data Records

The genomic PacBio sequencing, RNA-seq and Iso-seq data have been updated to the National Center for Biotechnology Information (NCBI) under the BioProject of PRJNA1015288^59^. *R. nymphaeae* genome assembly and the gene annotation have been deposited in Genbank under the accession number JAZAQC000000000^60^ and Figshare^61^.

## Technical Validation

We assessed the completeness and accuracy of *R. nymphaeae* genome assembly from five aspects. First, the summary statistics of the genome assembly revealed that the longest contig reaches 47.9 Mb, contig N50 reaches 12.7 Mb, and 38 contigs are longer than 1 Mb, constituting 98.45% of the assembly. All these data indicate that this assembly is one of the highest contiguous genome assemblies among 14 aphids that were in comparison (Supplementary Data 2). Second, the blob plots show that contaminant contigs, which were mainly from symbiotic bacteria, were completely removed from the assembly (Supplementary Figs. 1 and2). Third, using Merqury (v 1.3)^54^, we estimated the base-level accuracy and completeness of the *R. nymphaeae* assembly by comparing k-mers from the final assembly to those in the PacBio HiFi reads. Merqury reported a consensus quality (QV) of 69 and a completeness of 96.15% for the *R. nymphaeae* assembly, as visualized by the spectra-cn plot (Supplementary Fig. 3). In the spectra-cn plot, a homozygous peak was found at 90X coverage, suggesting a highly complete and accurate assembly. Fourth, the BUSCO evaluation indicated that 97.5% of gene orthologs (97% are single copy and 0.5% are duplicated) are present in the *R. nymphaeae* genome assembly (Supplementary Table 1). Lastly, the mapping rate of RNA-seq and Iso-seq reads are as high as 95.85% and 95.98%, respectively. These results, together, support our conclusion of a high-quality genome assembly.

Two methods were adopted to check the quality of protein-coding gene annotation in the *R. nymphaeae* genome assembly. First, BUSCO evaluation was used again, but this time under the “-m proteins” mode, and it suggested that the completeness of the annotation reached 95.3% (94.1% are single copy and 1.2% are duplicated, Supplementary Table 1). Second, DOGMA, a tool that assesses predicted proteins based on the conserved protein domains, indicated that 85.03% of conserved domains could be found in the *R. nymphaeae* gene annotation.

### Supplementary information

Supplementary information

Supplementary data 1

Supplementary data 2

## Data Availability

All the data analysis procedures were done following published manuals or public protocols of the software described in the Methods. Parameters for each software were detailed. Codes used to run gene annotation pipelines were deposited in GitHub with the link: https://github.com/Xu-lab-Evolution/Waterlily_aphid_genome_project.

## References

[1] Blackman, R. L. & Eastop, V. F. *Aphids on the world’s trees: an identification and information guide*. (Cab International, 1994).

[2] Ted D. Center, F. A. D. Jr., Greg P. Jubinsky, & Michael J. Grodowitz. *Insects and other arthropods that feed on aquatic and wetland plants* (United States Department of Agriculture) (Technical Bulletin, 1999).

[3] Braendle C (2003). Developmental origin and evolution of bacteriocytes in the aphid-Buchnera symbiosis. PLoS Biol..

[4] Wilson AC (2010). Genomic insight into the amino acid relations of the pea aphid, Acyrthosiphon pisum, with its symbiotic bacterium Buchnera aphidicola. Insect Mol. Biol..

[5] Nicholson SJ (2015). The genome of Diuraphis noxia, a global aphid pest of small grains. BMC Genomics.

[6] Thorpe P, Escudero-Martinez CM, Cock PJA, Eves-van den Akker S, Bos JIB (2018). Shared Transcriptional Control and Disparate Gain and Loss of Aphid Parasitism Genes. Genome Biol. Evol..

[7] Jiang, X. *et al*. A chromosome-level draft genome of the grain aphid Sitobion miscanthi. *Gigascience* **8** (2019). 10.1093/gigascience/giz101 PMC6701489 31430367

[8] Quan QM (2019). Draft genome of the cotton aphid Aphis gossypii. Insect Biochem. Mol. Biol..

[9] Mathers TC, Mugford ST, Hogenhout SA, Tripathi L (2020). Genome Sequence of the Banana Aphid, Pentalonia nigronervosa Coquerel (Hemiptera: Aphididae) and Its Symbionts. G3-Genes Genom. Genet..

[10] Wenger, J. A. *et al*. Whole genome sequence of the soybean aphid, Aphis glycines. *Insect Biochem. Mol. Biol*. **123** (2020). 10.1016/j.ibmb.2017.01.005 28119199

[11] Mathers TC (2021). Chromosome-Scale Genome Assemblies of Aphids Reveal Extensively Rearranged Autosomes and Long-Term Conservation of the X Chromosome. Mol. Biol. Evol..

[12] Wei, H. Y. *et al*. Chromosome-level genome assembly for the horned-gall aphid provides insights into interactions between gall-making insect and its host plant. *Ecol. Evol*. **12** (2022). 10.1002/ece3.8815 PMC9021935 35475184

[13] Stanke, M., Schoffmann, O., Morgenstern, B. & Waack, S. Gene prediction in eukaryotes with a generalized hidden Markov model that uses hints from external sources. *BMC Bioinformatics* **7** (2006). 10.1186/1471-2105-7-62 PMC1409804 16469098

[14] Stanke M, Diekhans M, Baertsch R, Haussler D (2008). Using native and syntenically mapped cDNA alignments to improve de novo gene finding. Bioinformatics.

[15] Hoff KJ, Lange S, Lomsadze A, Borodovsky M, Stanke M (2016). BRAKER1: Unsupervised RNA-Seq-Based Genome Annotation with GeneMark-ET and AUGUSTUS. Bioinformatics.

[16] Hoff KJ, Lomsadze A, Borodovsky M, Stanke M (2019). Whole-Genome Annotation with BRAKER. Gene Prediction: Methods and Protocols.

[17] Bruna, T., Hoff, K. J., Lomsadze, A., Stanke, M. & Borodovsky, M. BRAKER2: automatic eukaryotic genome annotation with GeneMark-EP plus and AUGUSTUS supported by a protein database. *NAR Genom. Bioinform*. **3** (2021). 10.1093/nargab/lqaa108 PMC7787252 33575650

[18] Gabriel, L., Hoff, K. J., Bruna, T., Borodovsky, M. & Stanke, M. TSEBRA: transcript selector for BRAKER. *BMC Bioinformatics* **22** (2021). 10.1186/s12859-021-04482-0 PMC8620231 34823473

[19] Park J, Kim Y, Xi H, Park J, Lee W (2020). The complete mitochondrial genome of Rhopalosiphum nymphaeae (Linnaeus, 1761) (Hemiptera: Aphididae). Mitochondrial DNA B Res..

[20] Chen, W. B. *et al*. Genome sequence of the corn leaf aphid (Rhopalosiphum maidis Fitch). *Gigascience* **8** (2019). 10.1093/gigascience/giz033 PMC6451198 30953568

[21] Cheng H, Concepcion GT, Feng X, Zhang H, Li H (2021). Haplotype-resolved de novo assembly using phased assembly graphs with hifiasm. Nat. Methods.

[22] Cheng H (2022). Haplotype-resolved assembly of diploid genomes without parental data. Nat. Biotechnol..

[23] Astashyn, A. *et al*. Rapid and sensitive detection of genome contamination at scale with FCS-GX. *bioRxiv*, 2023.2006. 2002.543519 (2023). 10.1186/s13059-024-03198-7 PMC10898089 38409096

[24] Laetsch DR, Blaxter ML (2017). BlobTools: Interrogation of genome assemblies. F1000Research.

[25] UniProt, C. (2019). UniProt: a worldwide hub of protein knowledge. Nucleic Acids Res..

[26] Buchfink B, Reuter K, Drost H-G (2021). Sensitive protein alignments at tree-of-life scale using DIAMOND. Nat. Methods.

[27] Flynn JM (2020). RepeatModeler2 for automated genomic discovery of transposable element families. Proc. Natl. Acad. Sci. USA.

[28] Altschul SF, Gish W, Miller W, Myers EW, Lipman DJ (1990). Basic local alignment search tool. J. Mol. Biol..

[29] Camacho C (2009). BLAST+: architecture and applications. BMC Bioinformatics.

[30] Li H (2009). The Sequence Alignment/Map format and SAMtools. Bioinformatics.

[31] Barnett DW, Garrison EK, Quinlan AR, Stromberg MP, Marth GT (2011). BamTools: a C++ API and toolkit for analyzing and managing BAM files. Bioinformatics.

[32] Lomsadze, A., Burns, P. D. & Borodovsky, M. Integration of mapped RNA-Seq reads into automatic training of eukaryotic gene finding algorithm. *Nucleic Acids Res*. **42** (2014). 10.1093/nar/gku557 PMC4150757 24990371

[33] Lomsadze A, Ter-Hovhannisyan V, Chernoff YO, Borodovsky M (2005). Gene identification in novel eukaryotic genomes by self-training algorithm. Nucleic Acids Res..

[34] Iwata, H. & Gotoh, O. Benchmarking spliced alignment programs including Spaln2, an extended version of Spaln that incorporates additional species-specific features. *Nucleic Acids Res*. **40** (2012). 10.1093/nar/gks708 PMC3488211 22848105

[35] Gotoh, O., Morita, M. & Nelson, D. R. Assessment and refinement of eukaryotic gene structure prediction with gene-structure-aware multiple protein sequence alignment. *BMC Bioinformatics* **15** (2014). 10.1186/1471-2105-15-189 PMC4065584 24927652

[36] Buchfink B, Xie C, Huson DH (2015). Fast and sensitive protein alignment using DIAMOND. Nat. Methods.

[37] Bruna, T., Lomsadze, A. & Borodovsky, M. GeneMark-EP plus: eukaryotic gene prediction with self-training in the space of genes and proteins. *NAR Genom. Bioinform*. **2** (2020). 10.1093/nargab/lqaa026 PMC7222226 32440658

[38] Bolger AM, Lohse M, Usadel B (2014). Trimmomatic: a flexible trimmer for Illumina sequence data. Bioinformatics.

[39] Kim D, Paggi JM, Park C, Bennett C, Salzberg SL (2019). Graph-based genome alignment and genotyping with HISAT2 and HISAT-genotype. Nat. Biotechnol..

[40] Stanke M (2006). AUGUSTUS: ab initio prediction of alternative transcripts. Nucleic Acids Res..

[41] Bateman A (2019). UniProt: a worldwide hub of protein knowledge. Nucleic Acids Res..

[42] Li H (2018). Minimap2: pairwise alignment for nucleotide sequences. Bioinformatics.

[43] Tang, S. Y. Y., Lomsadze, A. & Borodovsky, M. Identification of protein coding regions in RNA transcripts. *Nucleic Acids Res*. **43** (2015). 10.1093/nar/gkv227 PMC4499116 25870408

[44] Jones P (2014). InterProScan 5: genome-scale protein function classification. Bioinformatics.

[45] Morales-Hojas R (2020). Population genetic structure and predominance of cyclical parthenogenesis in the bird cherry-oat aphid Rhopalosiphum padi in England. Evol. Appl..

[46] Rispe C (2020). The genome sequence of the grape phylloxera provides insights into the evolution, adaptation, and invasion routes of an iconic pest. BMC Biol..

[47] Emms, D. M. & Kelly, S. OrthoFinder: solving fundamental biases in whole genome comparisons dramatically improves orthogroup inference accuracy. *Genome Biol*. **16** (2015). 10.1186/s13059-015-0721-2 PMC4531804 26243257

[48] Emms, D. M. & Kelly, S. OrthoFinder: phylogenetic orthology inference for comparative genomics. *Genome Biol*. **20** (2019). 10.1186/s13059-019-1832-y PMC6857279 31727128

[49] Darriba D (2020). ModelTest-NG: A New and Scalable Tool for the Selection of DNA and Protein Evolutionary Models. Mol. Biol. Evol..

[50] Kozlov AM, Darriba D, Flouri T, Morel B, Stamatakis A (2019). RAxML-NG: a fast, scalable and user-friendly tool for maximum likelihood phylogenetic inference. Bioinformatics.

[51] Letunic I, Bork P (2021). Interactive Tree Of Life (iTOL) v5: an online tool for phylogenetic tree display and annotation. Nucleic Acids Res..

[52] Wang, Y. P. *et al*. MCScanX: a toolkit for detection and evolutionary analysis of gene synteny and collinearity. *Nucleic Acids Res*. **40** (2012). 10.1093/nar/gkr1293 PMC3326336 22217600

[53] Bandi, V. & Gutwin, C. in *Graphics Interface 2020*.

[54] Rhie A, Walenz BP, Koren S, Phillippy AM (2020). Merqury: reference-free quality, completeness, and phasing assessment for genome assemblies. Genome Biol..

[55] Seppey M, Manni M, Zdobnov EM (2019). BUSCO: Assessing Genome Assembly and Annotation Completeness. Gene Prediction: Methods and Protocols.

[56] Dohmen E, Kremer LP, Bornberg-Bauer E, Kemena C (2016). DOGMA: domain-based transcriptome and proteome quality assessment. Bioinformatics.

[57] Kemena C, Dohmen E, Bornberg-Bauer E (2019). DOGMA: a web server for proteome and transcriptome quality assessment. Nucleic Acids Res..

[58] Diesh, C. *et al*. JBrowse 2: a modular genome browser with views of synteny and structural variation. *Genome Biol*. **24** (2023). 10.1186/s13059-023-02914-z PMC10108523 37069644

[59] (2024). NCBI Sequence Read Archive..

[60] Wang Y, Xu S (2024). Genbank.

[61] Wang Y, Xu S (2024). figshare.

