# Supplementary material for: A high-quality genome assembly of the waterlily aphid Rhopalosiphum nymphaeae
Source: Sci Data. 2024 Feb 13;11:194. doi: 10.1038/s41597-024-03043-3 (PMC10864314; doi:10.1038/s41597-024-03043-3)
Supplement: Supplementary file 1 — Supplementary information [file 41597_2024_3043_MOESM1_ESM.pdf]

Supplementary information for

'A high-quality genome assembly of the waterlily aphid *Rhopalosiphum nymphaeae*'

**Content**	**Page**
Supplementary Fig. 1	1
Supplementary Fig. 2	2
Supplementary Fig. 3	3
Supplementary Table 1	4

Supplementary Figures

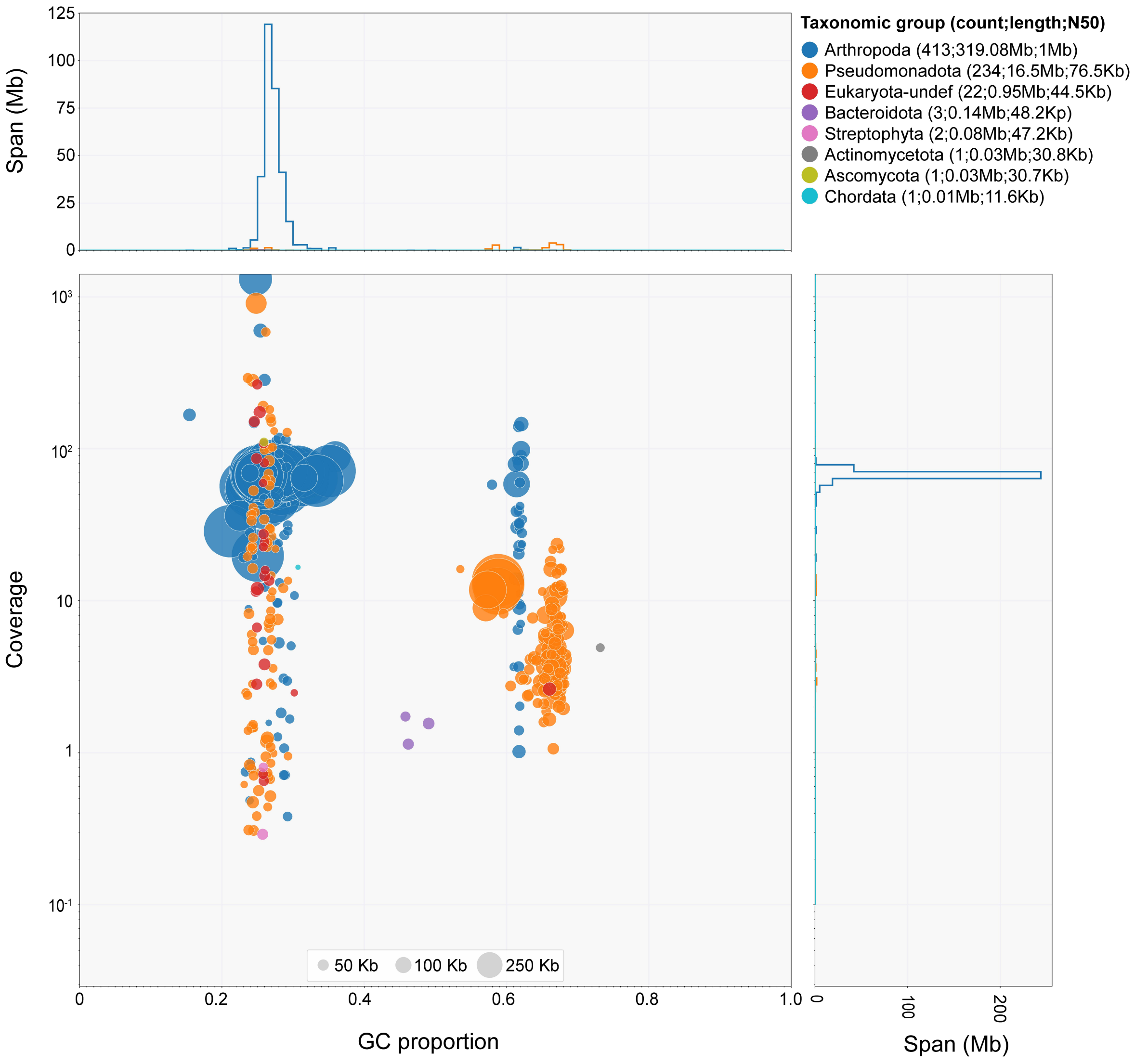

Supplementary Fig. 1. Blobplot of the initial *R. nymphaeae* assembly.

In the main scatter plot, circles represent assembled contigs, with their size being proportional to the contig length (assembly contigs longer than 1 Mb were split into smaller fragments of 1 Mb each). The Y-axis indicates read coverage, while the X-axis represents GC content proportion. The different colors of the circles correspond to their taxonomic information, as shown in the legend. Histograms displayed above and to the right of the main scatter plot illustrate the distribution of coverage and GC content proportion, respectively.

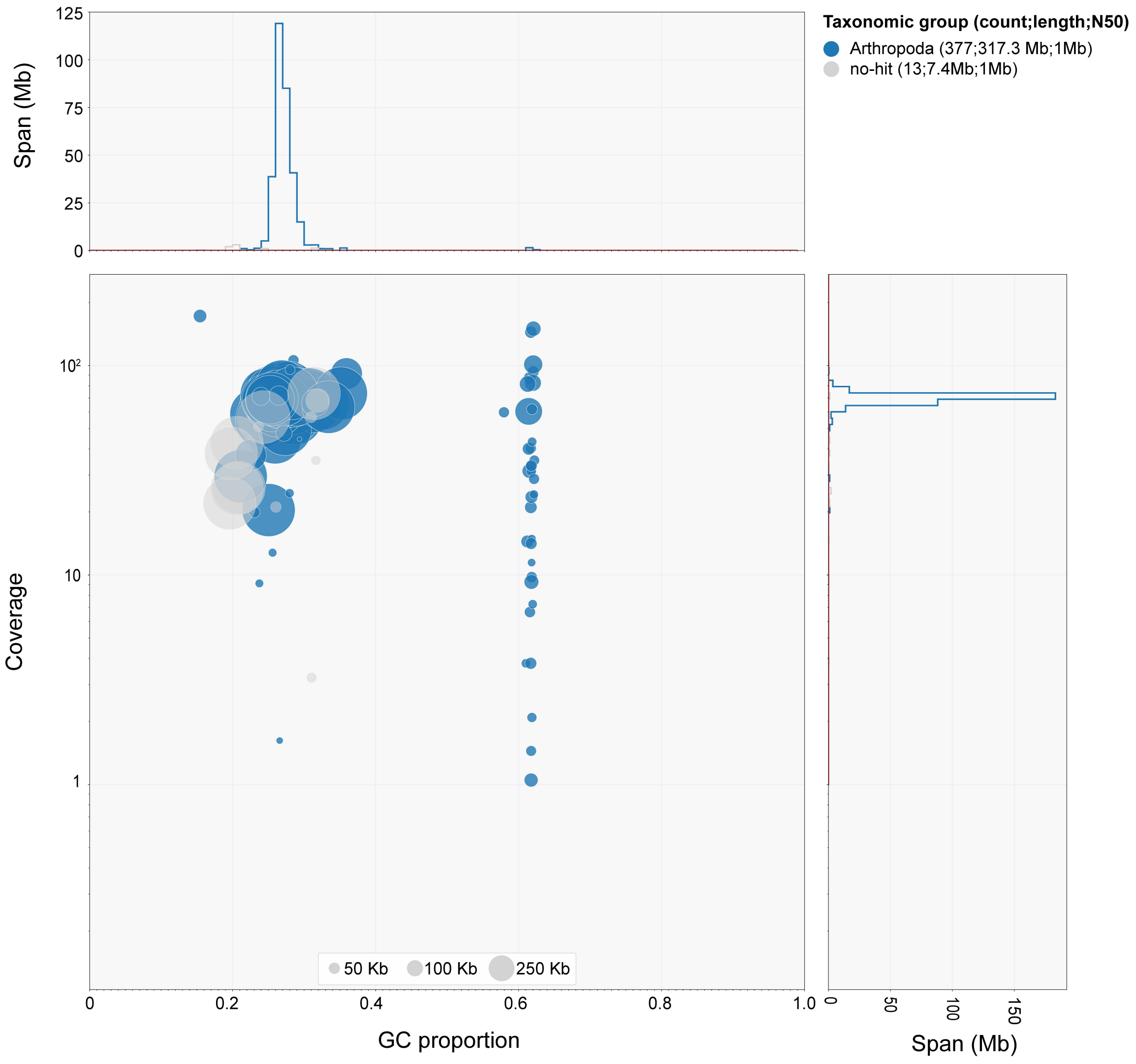

Supplementary Fig. 2. Blobplot of the final *R. nymphaeae* assembly (after removing the contaminants).

In the main scatter plot, circles represent assembled contigs, with their size being proportional to the contig length (assembly contigs longer than 1 Mb were split into smaller fragments of 1 Mb each). The Y-axis indicates read coverage, while the X-axis represents GC content proportion. The different colors of the circles correspond to their taxonomic information, as shown in the legend. Histograms displayed above and to the right of the main scatter plot illustrate the distribution of coverage and GC content proportion, respectively. The 'no-hit' contigs are those that did not show any mapping hits against the UniProt reference database. However, subsequent screening with FCS-GX confirmed that these contigs exhibit high similarity to *Rhopalosiphum maidis*, suggesting that they are unlikely to be contaminants.

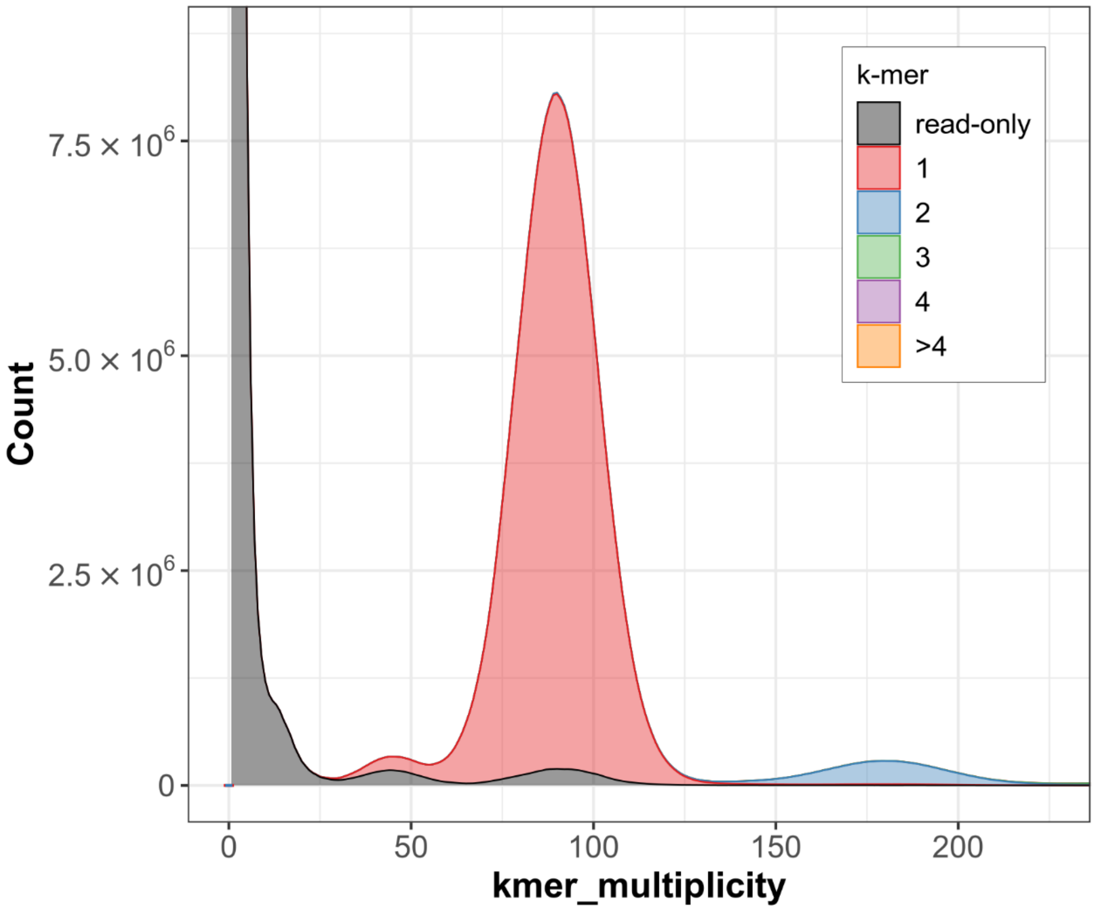

Supplementary Fig. 3. The kmer pectra-cn plot of the *R. nymphaeae* assembly.

The spectra-cn plot displays the multiplicity of each k-mer present in the PacBio HiFi read, using different colors to indicate its estimated copy number in the *R. nymphaeae* assembly. The X-axis represents the number of occurrences (multiplicity) of each k-mer, while the Y-axis shows the total count of different k-mers with that specific frequency.

Supplementary Table

Supplementary Table 1. BUSCO evaluations of genome assembly and annotation of Aphidini tribe with the "hemiptera_odb10" set of genes.

	References	Genome evaluation			Annotation evaluation		
		C (%)	F (%)	M (%)	C (%)	F (%)	M (%)
R. padi	Morales-Hojas R. *et al.*, 2020[1]	99.0[S:98.0,D:1.0]	0.4	0.6	98.2[S:97.1,D:1.1]	0.6	1.2
R. maidis	Chen *et al.*, 2019[2]	99.6[S:98.0,D:1.6]	0.2	0.2	95.5[S:93.3,D:2.2]	0.6	3.9
A. glycines	Wenger *et al.*, 2020[3]	97.7[S:94.7,D:3.0]	0.5	1.8	97.1[S:94.6,D:2.5]	0.6	2.3
A. gossypii	Quan *et al.*, 2019[4]	96.6[S:95.5,D:1.1]	1	2.4	96.7[S:94.0,D:2.7]	0.9	2.4
R. nymphaeae	This study	97.5[S:97.0,D:0.5]	0.3	2.2	95.3[S:94.1,D:1.2]	0.1	4.6

Note: C: Completeness; F: Fragmented; M: Missing; S: Single-copy; D: Duplicated.

References

1 Morales-Hojas, R. *et al.* Population genetic structure and predominance of cyclical parthenogenesis in the bird cherry-oat aphid *Rhopalosiphum padi* in England. *Evol. Appl.* **13**, 1009-1025 (2020).

2 Chen, W. B. *et al.* Genome sequence of the corn leaf aphid (*Rhopalosiphum maidis* Fitch). *Gigascience* **8** (2019).

3 Wenger, J. A. *et al.* Whole genome sequence of the soybean aphid, *Aphis glycines*. Insect Biochem. *Mol. Biol.* **123** (2020).

4 Quan, Q. M. *et al.* Draft genome of the cotton aphid *Aphis gossypii. Insect Biochem. Mol. Biol.* **105**, 25-32 (2019).
